# Zinc finger arrays binding human papillomavirus types 16 and 18 genomic DNA: precursors of gene-therapeutics for *in-situ* reversal of associated cervical neoplasia

**DOI:** 10.1186/1742-4682-9-30

**Published:** 2012-07-28

**Authors:** Misaki Wayengera

**Affiliations:** 1 Unit of Genetics, Genomics & Theoretical Biology, Dept of Pathology, School of Biomedical Science, College of Health Sciences, Makerere University, P. O. Box 7072, Kampala, Uganda

## Abstract

**Background:**

Human papillomavirus (HPV) types 16 and 18 are the high-risk, sexually transmitted infectious causes of most cervical intraepithelial neoplasias (CIN) or cancers. While efficacious vaccines to reduce the sexual acquisition of these high-risk HPVs have recently been introduced, no virus-targeted therapies exist for those already exposed and infected. Considering the oncogenic role of the transforming (E6 and E7) genes of high-risk HPVs in the slow pathogenesis of cervical cancer, we hypothesize that timely disruption or abolition of HPV genome expression within pre-cancerous lesions identified at screening may reverse neoplasia. We aimed to derive model zinc finger nucleases (ZFNs) for mutagenesis of the genomes of two high-risk HPV (types 16 & 18).

**Methods and results:**

Using ZiFiT software and the complete genomes of HPV types16 and 18, we computationally generated the consensus amino acid sequences of the DNA-binding domains (F1, F2, & F3) of (i) 296 & 327 contextually unpaired (or single) three zinc-finger arrays (sZFAs) and (ii) 9 & 13 contextually paired (left and right) three- zinc-finger arrays (pZFAs) that bind genomic DNA of HPV-types 16 and 18 respectively, inclusive of the E7 gene (s/pZFA_HpV/E7_). In the absence of contextually paired three-zinc-finger arrays (pZFAs) that bind DNA corresponding to the genomic context of the E6 gene of either HPV type, we derived the DNA binding domains of another set of 9 & 14 contextually unpaired E6 gene-binding ZFAs (sZFA_E6_) to aid the future quest for paired ZFAs to target E6 gene sequences in both HPV types studied (pZFA_E6_). This paper presents models for (i) synthesis of hybrid ZFNs that cleave within the genomic DNA of either HPV type, by linking the gene sequences of the DNA-cleavage domain of the *FokI* endonuclease F_N_ to the gene sequences of a member of the paired-HPV-binding ZFAs (pZFA_HpV/E7_ + F_N_), and (ii) delivery of the same into precancerous lesions using HPV-derived viral plasmids or vectors.

**Conclusions:**

With further optimization, these model ZFNs offer the opportunity to induce target-mutagenesis and gene-therapeutic reversal of cervical neoplasia associated with HPV types 16 & 18.

## Background

### High-risk human papillomaviruses (HPVs) as oncogenic agents associated with cervical neoplasia

Human papillomaviruses (HPVs) are circular double stranded (ds) deoxyribonucleic acids (DNA) viruses of the genus *alphapapillomavirus* and family *papillomavirus*. Productive infection with HPVs only occurs within the stratified epithelium of the skin or mucous membranes [1]. Although there are over 200 HPV types, and 30 to 40 of these are sexually transmitted, only types 16, 18, 31, 33, 35, 39, 45, 51, 52, 56, 58 and 59 are denoted ‘high-risk’ HPVs [2,3]. Infection with these HPV types may lead to the development of cervical, penile or anal intraepithelial neoplasia. This is because most HPV infections are temporary, being eventually cleared by the immune system (70% and 90% in the first and second year following infection, respectively) with little or no long-term clinical significance [4]. In some individuals (representing 5% to 10% of infected women), however, the infection persists and could increase the risk for development of pre-cancerous lesions of the cervix, which can progress to invasive cervical cancer [4]. Cervical cancer is the cause of substantial morbidity and mortality among women world-wide. Each year, an estimated 490,000 cases of cervical cancer occur, resulting in approximately 270,000 deaths [1,2]. High-risk HPVs induce malignant transformation (neoplasia) and promote tumor growth through their early oncogenes E6 (HpVgp1) and E7 (HpVgp2). On the one hand, E6, which has a close relationship with the cellular protein E6-AP (E6-associated protein), mediates ubiquitin binding to the p53 protein, thereby flagging it for proteosomal degra-dation [5]. Degradation of p53, a protein that primarily prevents cell growth and stimulates apoptosis in the presence of DNA damage, promotes neoplasia. When present in normal levels, p53 also upregulates the p21 protein, which blocks the formation of the cyclin D/Cdk4 complex. This prevents the phosphorylation of retinoblastoma protein (pRB) and in turn halts cell cycle progression by preventing the activation of the eukaryotic transcription factor E2F. In short, p53 is either absent or its levels are greatly reduced within cervical cancer cells [6,7]. On the other hand*,* the E7 oncoprotein induces neoplasia by binding to and acting on multiple functional partners, notably retinoblastoma protein (pRB) and the class I histone deacetylases HDAC1 and HDAC2 [8-10]. Specifically, E7 destabilizes pRB levels through cullin 2–mediated proteasomal degradation [8]. E7 also indirectly binds to HDAC1 and HDAC2 as well as the reverse transcriptase (hTERT) region of telomerase via sequences in its zinc-finger domain [9]. While mutations within the zinc-finger domain of E7 do not affect its propensity to bind to and degrade pRB, they do abrogate its ability to immortalize cells, suggesting that both activities of E7 are required for immortalization [7-10]. Overall, cervical cancer cells can be said to be addicted to and or to thrive on the expression of the E6 and E7 proteins of high-risk HPVs.

### Limitation of existing treatment and prevention modalities for ‘high-risk’ HPV

The slow neoplastic process following infection with high-risk HPVs, which usually takes 15–20 years, provides many opportunities for early detection and treatment of the pre-cancerous lesion. Specifically, progression to invasive cancer can almost always be prevented when standard prevention strategies are applied. In most countries, cervical screening using a Papanicolaou (Pap) test [11] or liquid-based cytology [12] is used to detect abnormal cells that could develop into cancer. In addition, targeted screening for HPVs or diagnostic testing (detecting E6, E7, or p16 mRNA) is now available [13-15]. If and when abnormal cells are found, women are invited to have a colposcopy with visual inspection under acetoacetic acid (VIUA). During a colposcopic inspection, biopsies can be taken and abnormal areas removed with simple procedures, typically by wedge- biopsy, cauterizing loop, freezing (cryotherapy), or chemo-/radio-therapy [16]. Treating abnormal cells in this way can halt the initiated development of cervical cancer [17]. However, only a few high-risk women, particularly within the developing world, ever get the timely test to detect abnormal cervical lesions. In addition, only a minority of those who are tested and are positive ever receive the therapy required to arrest disease. This may be attributed to inadequate follow-up and poor referral systems within poorly-resourced country health centers. Recent development of HPV vaccines (Cervarix and Gardasil), which when effectively used can prevent primary infection with the HPV types (16 and 18) that cause 70% of cervical cancers, has generated optimism that the global incidence of cervical cancer may be subdued [18,19]. It is nevertheless important to acknowledge that (i) coverage of HPV vaccination remains low within resource-limited yet cervical cancer-high incidence settings, and (ii) the current HPV vaccines are only effective when offered to girls who are not yet sexually active and have therefore never been exposed to high-risk HPVs.

Overall, the picture described above underscores the relevance of investing in development of specifically HPV-targeted treatments as a complementary public health strategy for controlling the global incidence of cervical cancer. More recently, Lin et al. [20] have proposed and experimentally tested the use of proteosome and histone de-acetylase (HDAC) inhibitors as novel HPV-targeted treatments for cervical cancer.

### The alternate option of directly disrupting or abolishing HPV gene expression

Bacteria endowed with the restriction modification (R-M) system exhibit amazing resistance to tropism by the xenogeneic DNA of bacteria-infecting viruses, bacteriophages. RM systems can be said to be a primitive form of the many restriction factors that have recently been found to possess innate antiviral properties [21-23]. I [21] previously proposed using the anti-phage DNA machinery inherent within bacterial RM systems as a model for devising eukaryotic virus gene therapies. Towards this goal, I and colleagues [22,23] identified several bacterially-derived restriction enzymes with potential to cleave the DNA of human-infecting viruses, including frequency and site mapping of HIV-1, HIV-2 and several other SIV gene cleavages using a proviral DNA model [23]. However, most naturally-occurring bacterial restriction enzymes (aka REases) previously found to possess high potency (to slice or disrupt) against human infecting viruses in this manner were observed to cleave at palindromic sites that are short and equally prevalent within the human host genome, thereby raising concerns about *in-situ* safety. Our failure to address this priority milestone limited the use of those REase-therapeutic precursors to modeling and the extracellular space [22]. The long-term goal in the field of RM enzymatic therapeutics has been to generate synthetic restriction endonucleases with longer recognition sites specific only for the eukaryotic virus by mutating or engineering existing enzymes. Zinc finger nucleases (ZFNs) are artificial, hybrid restriction enzymes created by covalently linking a DNA-binding zinc finger domain (composed of three to six finger arrays) to the non-specific DNA cleavage domain (or simply F_N_) of the *Flavobacterium okeanokoites* restriction endonuclease *FokI*[24]. ZFNs have recently emerged as a potentially powerful tool for fighting eukaryotic viruses, either by primarily editing host genomes, or secondarily targeting incoming viral genomes [24-32]. On the one hand, Perez et al. [29], using engineered ZFNs targeting human CCR5, previously demonstrated the establishment of HIV-1 resistance in CD4+ T cells through generation of a double-strand break (DSB) at predetermined sites in the CCR5 coding region upstream of the natural CCR5D32 mutation. More recently, Holt et al. [30] demonstrated control of HIV-1 infection within NSG-mice transplanted with human hematopoietic stem/progenitor cells modified by zinc-finger nucleases targeting CCR5. On the other hand*,* with the intent of disrupting incoming viral genomes, we recently identified DNA binding domains of ZFNs to use as gene-therapeutics against proviral HIV DNA and HSV-II [31,32].

In the light of the above literature, we hypothesized that target mutagenesis of those high-risk forms of HPVs using ZFNs may possess potential for genetic therapy and reversal of cervical cancer *in-situ.*

The aim of this study was to identify and model DNA-binding domains of zinc finger arrays (ZFAs) as precursors for (i) synthesizing ZFNs for target mutagenesis of two high-risk HPV (types 16 & 18) –genomes, and development of a novel gene-therapeutic armament for reversing the primary oncogenic processes leading to cervical neoplasia.

## Methods and results

### (a) Database of DNA-binding domains of contextually unpaired three zinc-finger arrays targeting HPV types 16 and 18 genomic DNA

**Figure 1:**
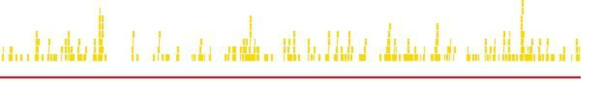
**Schematics of zinc finger arrays targeting HPV type 16 genomic DNA.** This figure offers a diagrammatic illustration of loci of action of zinc finger arrays that specifically bind to 9 bp nucleotide sequences within the genomic DNA context of HPV type 16. Hits (blue, green, and gold bars) represent targets along the gene (red bars). ZFAs hits in the graphic are color-coded on the basis of spacer size (5 bp = Blue; 6 bp = Green; 7 bp = Gold).

**Figure 2:**
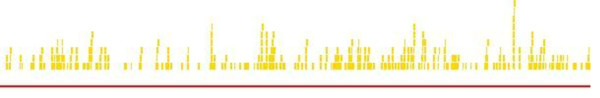
**Schematics of zinc finger arrays binding to HPV type 18 genomic DNA.** This figure offers a diagrammatic illustration of loci of action of zinc finger arrays that specifically bind to 9 bp nucleotide sequences within the genomic DNA context of HPV type 18. Hits (blue, green, and gold bars) represent targets along the gene (red bars). ZFA hits in the graphic are color-coded on the basis of spacer size (5 bp = Blue; 6 bp = Green; 7 bp = Gold).

First, using *Context-Dependent Assembly (CoDA)* inherent in the ZiFiT-ZFA software of the zinc finger consortium [33-36] and the complete genomes of HPV types 16 and 18, we computationally generated the consensus amino acid sequences of the DNA binding domains or F1, F2 & F3 helices of 296 (see, Additional file 1 and Figure 1) and 327 (see, Additional file 2 and Figure 2) unpaired (single, either left or right) three zinc- finger (Zif) arrays (sZFAs) for targeting genomic DNA of HPV types 16 and 18, respectively. These ZFAs are henceforth denoted sZFA_HpV16_ and sZFA_HpV18_ respectively, or simply sZFA_HpV_. In principle, the FASTA formats of each virus type’s complete genomic DNA sequences (NCBI Accession #: |NC_001526.2| and |NC_001357.1| or IDs: 5607 and 5608, respectively) were individually fed into the user-interface preset to assemble three finger arrays. Although the HPV genomes do not contain intronic sequences, the exon/intron case-sensitivity algorithm was turned to its ON-mode throughout these experiments; this algorithm is necessary to distinguish between intron and exon sequences by denoting exons in uppercase and introns in lowercase. Overall, for either virus type, we derived DNA-binding domains of unpaired or single three-ZFAs (sZFAs) capable of specifically recognizing and binding to consecutive 9 bp sequences across the entire genome (see Figures 1 and 2, respectively). Analyses of the issuing cleavage patterns revealed that the highest incidence of unpaired ZFA-binding HPV type 16 genomic DNA is situated within the first and last 1,581 bp (20% of 7,905 bp) of the complete HPV type 16 genome (genomic context: before 0.2 and after 0.8, respectively) (see Figure 1). According to Zheng and Baker [37], the HPV genome can be organized into three major regions: early, late, and a long control region (LCR or non-coding region [NCR]), which are separated by two polyadenylation (pA) sites: early pA (A_E_) and late pA (A_L_). The early region of a papillomavirus genome occupies over 50% of the genome from its 5′ end and encodes six common open reading frames (E1, E2, E4, E5, E6 and E7) that translate their respective proteins. Two additional ORFs, E3 and E8, were assigned to this region initially. However, only the E8 ORF in bovine papilloma virus-1 and HPV-31 have so far been proven to encode a spliced E8^E2C fusion protein, which functions as a negative regulator of viral transcription and replication. The late region of every papillomavirus genome, covering almost 40% of the genome, lies downstream of the early region and encodes L1 and L2 ORFs for translation of a major (L1) and a minor (L2) capsid protein. The long control region (LCR), a segment of about 850 bp (10% of the HPV genome), has no protein-coding function, but bears the origin of replication as well as multiple transcription factor binding sites that are important in regulation of RNA polymerase II-initiated transcription from viral early as well as late promoters. On the basis of this genome structure, those ZFAs binding to the 5′ end 20% would fall under the early region, while the 3′ end 20% falls both under the late and LCR region. For HPV type 18, on the other hand, despite an occurrence of ZFAs with DNA-binding domains able to target 9 bp sequences distributed randomly across almost all its genome, the highest incidence of ZFAs with HPV-type 18 genomic DNA binding-domains was found to be situated within the last 1,571 bp (20% of the total genomic 7,857 bp) of the complete genome (genomic context: 0.8-1.00) (see Figure 2). In the context of the HPV genome structure provided by Zheng and Baker [37], this 3′ end falls under the late and long control region (LCR) of all papillomavirus genomes, and encodes L1 and L2 ORFs for translation of a major (L1) and a minor (L2) capsid protein, alongside the LCR regional origin of replication plus multiple transcription factors [37]. Each of these contextually unpaired or single zinc finger arrays (sZFAs) is composed of three zinc fingers (Zif or simply ZF). Zifs or ZFs are protein motifs that each possesses two beta strands and an alpha helix stabilized by coordination of a zinc ion mediated by pairs of conserved cysteine and histidine (C_2_H_2_) residues [38-40]. Residues 1 to 6 (numbered relative to the start) of the alpha-helix mediate target binding and recognition of triplets of DNA sequences through the formation of base-specific contacts in the major groove of the double-stranded target DNA (for illustration, see Additional file 1 and Additional file 2) [38-41]. Several Zifs/ZFs can be linked together, as is the case in the sZFA_HpV16_ and sZFA_HpV18_ databases, in order to yield a contextually unpaired multi-finger array capable of recognizing a longer and thereby preferentially unique sequence in any target double-stranded genomic DNA, aside from the host. As already shown in Additional file 1 and Additional file 2, several such contextually unpaired ZFAs were uncovered with target-binding potency across the entire genomic contexts of either HPV type 16 or 18 DNA.

### (b) Database of DNA-binding domains of contextually paired three zinc-finger arrays targeting HPV types 16 and 18 genomic DNA specific zinc finger nucleases

**Figure 3:**
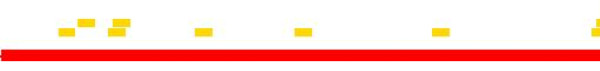
**Schematics of zinc finger nucleases cleaving HPV type 16 genomic DNA.** This figure offers a diagrammatic illustration of loci of action of zinc finger nucleases that target and cleave >18 bp (9x2 + 5, 6, or 7) sequences within the genomic DNA context of HPV type 16. Hits (blue, green, and gold bars) represent targets along the gene (red bars). ZFN hits in the graphic are color-coded on the basis of spacer size (5 bp = Blue; 6 bp = Green; 7 bp = Gold).

**Figure 4:**
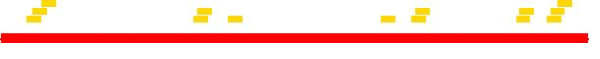
**Schematics of zinc finger nucleases cleaving HPV type 18 genomic DNA.** This figure offers a diagrammatic illustration of loci of action of zinc finger nucleases that target and cleave >18 bp (9x2 + 5, 6, or 7) sequences within the genomic DNA context of HPV type 18. Hits (blue, green, and gold bars) represent targets along the gene (red bars). ZFN hits in the graphic are color-coded on the basis of spacer size (5 bp = Blue; 6 bp = Green; 7 bp = Gold).

Second, using *Context-Dependent Assembly (CoDA)* inherent in the ZiFiT-ZFN software of the zinc finger consortium [33-36] and the complete genomes of HPV types 16 and 18, we computationally compiled the amino acid sequences of the alpha-helical DNA-binding domains of 9 and 13 paired three zinc-finger arrays targeting HPV types 16 and 18 genomic DNA, respectively (see Additional file 3 and Additional file 4, respectively). Throughout our assembly of the DNA-binding domains of these paired ZFA (pZFAs), all ZiFiT-ZFN algorithms were pre-set as they were for derivation of the unpaired ZFAs (sZFAs) above, except that a 5, 6, or 7 base-pair (bp) overlapping sequence was selected in addition. Because ZFNs function as dimers, it is these paired ZFAs (pZFAs) assembled in this section of the results that are intended for engineering ZFNs that cleave the genomes of the study HPV types, as modeled further below [38]. These paired ZFA (pZFAs) are henceforth denoted pZFA_HpV16_ and pZFA_HpV18_ respectively, or simply pZFA_HpV_. Overall, pZFA_HpV_ with demonstrable *in-silico* ability to bind to target sequences at positions 0.45 (~3,557 bp), 0.75 (~5,929 bp), and across 0.85 to 0.90 within the HPV type 16 genomic DNA context were derived. These genomic contextual regions approximately correspond to sequences between the early region’s hypothetical protein HpV16gp5 (context 3332-3619 bp) and the late region’s major L1 capsid protein (5,560-7,155 bp) (see Figure 3). In contrast, pZFA_HpV_ capable of binding at positions 0.1 (~786 bp), 0.25 (~1,964 bp), 0.45 (~3, 535 bp), 0.65 (~5,107 bp), 0.75 (~5,892 bp) and 0.85 (~6,679 bp) respectively within the HPV type 18 genomic DNA context were derived. These regions correspond to the genomic contexts of the genes E7, E1, E2, E3, E4, L2 and L1, respectively (see Figure 4). It is important to note that, while we have generated pZFA_HpV_ that are precursors for synthesizing HPV-specific ZFNs, engineering of the actual ZFNs can only be achieved *in-vitro* as is further modeled below (see section d of results).

### (c) Repository of DNA-binding domains of unpaired zinc finger arrays targeting HPV types 16 and 18 E6 genes

**Figure 5:**
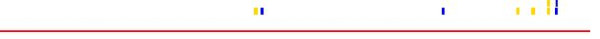
**Schematics of zinc finger arrays binding to HPV type 16 E6 gene.** This figure offers a diagrammatic illustration of loci of action of zinc finger arrays that specifically bind to 9 bp nucleotide sequences within the DNA context of the HPV type 16 transforming gene, E6. Hits (blue, green, and gold bars) represent targets along the gene (red bars). ZFA hits in the graphic are color-coded on the basis of spacer size (5 bp = Blue; 6 bp = Green; 7 bp = Gold).

**Figure 6:**
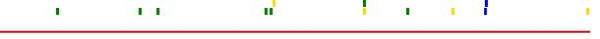
**Schematics of zinc finger arrays binding to HPV type 18 E6 gene.** This figure offers a diagrammatic illustration of loci of action of zinc finger arrays that specifically bind to 9 bp nucleotide sequences within the DNA context of the HPV type 18 transforming gene, E6. Hits (blue, green, and gold bars) represent targets along the gene (red bars). ZFA hits in the graphic are color-coded on the basis of spacer size (5 bp = Blue; 6 bp = Green; 7 bp = Gold).

Lastly*,* in the absence of any paired ZFA (pZFAs) that are precursors for engineering ZFNs targeting within the genomic context corresponding to the E6 transforming gene of either HPV type 16 (>gi|310698439:83-559|) or 18 (>gi|9626069:105–581), we focused our efforts on assembling another two databases of 9 (Figure 5) and 14 (Figure 6) DNA-binding domains of unpaired or single ZFAs (sZFAs) that target this early gene variant from HPV types 16 and 18, respectively. These sZFA databases offer us a basis for creating novel E6-targeting pZFAs precursors of ZFNs, by (i) sequential pairing of those neighboring sZFAs lying within the context of a desired pZFA target, and (ii) *in-vitro* optimization [42]. Alternatively, those E6 gene binding sZFAs may be used to enhance delivery of existing HPV-targeted treatments for cervical cancer such as the proteosome and histone deacetylase (HDAC) inhibitors previously described by Lin et al. [20].

### (d) Models for synthesis and delivery of ZFNs targeting HPV types 16 and 18

The following text describes the generation of models for (i) synthesis of hybrid ZFNs to cleave within either HPV type’s genomic DNA by linking the gene sequences of the DNA-cleavage domain of the *FokI* endonuclease-F_N_ to gene sequences coding for members of the paired-HPV-binding ZFAs (pZFA_HpV/E7_ + F_N_) and (ii) delivery of the same into precancerous lesions using HPV-derived viral plasmids or vectors:

First, using an approach similar to that described by Kim et al. (1996), employing the gene sequences of the DNA-cleavage domain of the *Fok I* endonuclease-F_N_ (derived from *Flavobacterium okeanokoites* and belonging to the type IIS class) and members of a pair of ZFAs (pZFAs) (see Additional file 3 and Additional file 4, respectively) in our databases provided in section b above, it is possible to fuse the two sequences to yield 9 and 13 hybrid, chimeric ZFNs (pZFA_HpV_ + F_N_) with ability to cleave the genomic DNAs of HPV types 16 and 18, respectively (for the predicted corresponding ZFN-cleavage sites, see Figures 3 and 4) [38]. The two individual components of the model ZFNs (pZFA_HpV_ and F_N_) are molecularly cloned into ZFN expression vectors *in-vitro* using unique XbaI/NotI restriction sites [33-36,38-41]. Specifically, PCR amplification of gene-sequences of each individual component is done using primers that introduce XbaI/NotI sites as a strategy to enable a pair of the pZFA_HpV_ + F_N_ complex to be inserted into alternative sites of Zinc Finger Consortia plasmids capable of recognizing target sites with a 7 bp spacer [33,34]. In principle, since the desired effect of the ZFN is achieved *in-vivo* by two paired zinc finger arrays (pZFAs) each fused to a nuclease domain, two members of a pair (pZFAs) are sub-cloned. Dimerization of the FokI nuclease occurs *in-vivo* when both members of the pZFA bind their target sequence, thereby ensuring that the two FokI nucleases attach to the target DNA in a particular configuration in order to introduce a double strand break (DSB). Following actual synthesis, it may be necessary to incorporate further improvements, say by (i) modular analysis to add one, two or even three other ZFA on to our currently three-paired ZFAs (pZFAs) so as to enhance specificity and avoid off-target genome toxicity [33-36]. *In-vitro* assembly and testing for efficacy of the desired target-cleavage need to be done pre-clinically say by (ii) using either a bacteria-one hybrid (B1H) or yeast one-hybrid (Y1B) system, so as to inform and select the best ZFNs to use *in-vivo*[42]. Elsewhere, (iii) the Fok1 cleavage domain has been modified as a strategy for generating a hybrid capable of functionally interrogating the ZFN dimer interface so as to prevent homodimerization while still enhancing the efficiency of cleavage [42].

*Second,* an exact approach toward gene delivery and transduction of the ‘pre-cancerous lesion’ cells with these ZF_HpV_ + F_N_ complexes* in-vivo *is theoretically proposed. Specifically, we propose the use of human papillomavirus plasmids capable of episomal replication in human cell lines, to deliver the diploid or paired copy of the high-risk HPV-specific ZFN genotype, pZF_HpV_ + F_N_. The pZF_HpV_ + F_N_ genotype may be sub-cloned using similar PCR-primers to the ones proposed above for consortia plasmids, with tailored modifications to HPV plasmids or PsV. Sverdrup et al. [43] have previously generated such human papillomavirus (HPV) plasmids containing the viral E1 and E2 genes (or the E1 gene alone) and an origin of replication, which they simultaneously demonstrated to replicate to significant levels in the transfected human cervical carcinoma C-33A cell line. Alternatively, HPV PsV encapsidation of the zinc finger consortium’s plasmids carrying the pZF_HpV_ + F_N_ (see results of modeling above) may suffice. Graham et al. [44], using such HPV PsV-encapsidation of DNA plasmids, produced mucosal vaccine vectors expressing an experimental antigen derived from the M and M2 proteins of respiratory syncytial virus. The same were shown to evoke 10,000-fold higher CD8+ T-cell and antibody responses in mice than naked DNA. Moreover, on the basis of light emission and immunofluorescence microscopy, it was shown by immunization with HPV PsV-encapsidated luciferase- and red fluorescent protein (RFP)-expressing plasmids that the HPV PsV-encapsidated plasmids induce antigen expression restricted to the vaginal epithelium (an ideal target), although this expression is only transient and further modifications of these HPV plasmids are needed to ensure stable expression of the exogenous pZF_HpV_ + F_N_ genotype [45].

### (e) Software and databases

The National Center for Biotechnology Information (NCBI) HPV type 16 and 18 genome databases used are available at the following URLs:

• http://www.ncbi.nlm.nih.gov/genome?term=NC_001526.2

• http://www.ncbi.nlm.nih.gov/Taxonomy/Browser/wwwtax.cgi?mode=Info&id=333761&lvl=3&lin=f&keep=1&srchmode=1&unlock

The two Zinc Finger Consortium’s CoDA-ZiFiT software used are available at the following URL: http://zifit.partners.org/ZiFiT/ChoiceMenu.aspx.

## Discussion

I present here databases of paired ZFAs (pZFA) that are precursors for engineering ZFNs to target and cleave the genomic DNA of the two high-risk HPV types most associated with cervical cancer. With the appropriate *in-vivo* gene-delivery and transduction plasmids or vectors, models of ZFNs synthesized from these pZFA may offer us the means for targeted HPV mutagenesis and therapeutic reversal of the primary oncogenic processes driving cervical neoplasia. Considering the oncogenic role of the transforming genes (E6/HpVgp1 or E7/HpVgp2) of high-risk types of HPVs in the slow pathogenesis of cervical cancer, therefore, we hypothesized that timely *in-situ* disruption or abolition of HPV genome expression within detected high-risk lesions may reverse cervical neoplasia. To this end, we identified DNA-binding domains of ZFAs for engineering ZFNs for targeted mutagenesis of these two high-risk HPV genomes as precursors for developing a novel gene-therapeutic armament for reversing the primary oncogenic processes leading to cervical neoplasia. As shown in Additional file 1 and Additional file 2, plus Additional file 3 and Additional file 4, respectively, multiple unpaired/single and paired ZFAs (Figures 1, 2, 3 and 4) were initially identified. The paired ZFAs (pZFAs) were used to model ZFNs that bind and cleave HPV type 16 and 18 genomic DNA (Figures 3 and 4). Because the modeled ZFNs could only target sequences of the E7 gene of *either* virus type studied, we focused our additional efforts on the E6 transforming gene, resulting in another set of two databases of 9 and 14 single ZFAs (sZFAs) that bind to sequences of this early gene from both types of HPV studied (16 and 18, respectively) (Figures 5 and 6).

Many previous studies have explored the therapeutic potential of ZFN technology against DNA viruses that infect humans, primarily through editing host genomes [27-30], secondarily by targeting the infective viral genomes [31,32]. ZFNs generally act by introducing a DSB into the target (say, HPV) genome which in the absence of a template for homologous recombination (HR) would lead to repair, if any, by non-homologous end-joining (NHEJ) [38-41]. NHEJ is therefore a major mechanism by which our modeled ZFNs are expected to induce gene architectural disruptions and functional distortions in the HPVs, although complete HPV gene deletion through frame-shift mutations is another possibility [33-36,38-41,46]. Towards a similar purpose [31,32], our model HPV-binding ZFNs would be usable first to cleave and disrupt HPV type 16 genomic DNA at the contextual positions corresponding to about 0.45 (~3,557 bp), 0.75 (~5,929 bp), and across 0.85 to 0.90. Disabling of these regions, which correspond to sequences between the early region’s hypothetical protein HpV16gp5 (context 3332-3619 bp) and the major L1 capsid protein (5,560-7,155 bp) (see Figure 3), may abrogate or reduce HPV type 16 survival or replication fitness. In contrast, HPV type 18 would be cleaved at regions approximately corresponding to the genomic contextual positions 0.1 (~786 bp), 0.25 (~1,964 bp), 0.45 (~3, 535 bp), 0.65 (~5,107 bp), 0.75 (~5,892 bp) and 0.85 (~6,679 bp); or simply the early-gene region (E7, E1, E2, E3, E4), late region (L2 and L1) and the LCR region (containing the origin of replication and multiple transcriptional factors) is predicted to be especially susceptible to similar abrogation or reduction in survival and replication fitness (Figure 4) [37]. Considering the addictive and oncogenic role of the HPV genes E6 and E7 in the pathogenesis of cervical dysplasia and/or neoplasia [6-10,20], it should be therapeutically adequate to target only these genes, explaining our further exploration of single ZFAs (sZFAs) for the E6 gene of either HPV type studied (Figures 5 and 6). Secondly*,* any two of the modular ZFNs cleaving at the extreme 5′ and 3′ ends of a viral genome could further be modified and optimized [42] to non-specifically target and delete most (i.e. >90%) of the genomic DNAs of the HPVs studied. In view of the currently evidenced low transduction and genome-modification rates of existing vectors and ZFN-technology, it is important that the success rates of effecting such changes in HPVs within precancerous lesions of the cervix are evaluated* in-vitro* and *in-vivo*, say by using HeLa cell lines or humanized mouse models [42,47-49]. Thirdly, it is rational to propose use of those single HPV-genome targeting ZFAs (sZFA_HpV_) as magnetic drivers for novel HPV-DNA targeting therapeutics such as transcriptional repressors or the proteosomal/histone deacetylase (HDAC) inhibitors discussed by Lin et al. [20].

This study has a number of limitations. First, the work has been limited to sequence analyses and is not accompanied by* in-vitro *studies. This can be attributed to the limited resource capacity of our laboratory. However, these same methods [33-36] have previously been employed successfully to assemble pZFAs that were used to engineer ZFNs, which have been experimentally proven to be safe and effective [33-44,46]. However, preclinical studies* in-vitro *using either a bacterial or yeast hybrid system as described in the modeling results in section d of our results need to be conducted to determine the efficacy of the model ZFN_HpV_ before their trial-use in the clinic. Experiments such as those recently conducted by Wilen et al. [47] for the Ad5/F35 vector carrying CXCR4-specific zinc-finger nucleases used to engineer HIV-resistant human CD4+ T cells may be conducted in humanized mouse models. Secondly, inclusion of ZFAs and ZFNs targeting other high risk HPVs, say types 31 and 45, may be a necessary strategy to increase the projected efficacy of this method from 70% (contribution of HPV types 16 and 18 to global cervical dysplasia) to nearly 100%. Thirdly*,* it is unlikely that the entire genomes of these high-risk HPVs will be excised through frame-shift mutations. Therefore, the already error-prone NHEJ-repair of the DSBs introduced in the HPV genomes could facilitate rare and random yet possibly effective recombinations that may yield either wild-type or mutant HPVs still capable of infecting and transforming cervical epithelia to cancer. This further underlines the need for the above proposed safety and efficacy studies. Fourthly, it must be asked how these modeled HPV-plasmids carrying and transducing ZFNs targeting HPV types 16 and 18 will be used in the clinic. Our translational projection or proposition is to achieve this via direct subdermal or intra-epithelial injection into those sites identified by currently existing screening protocols as pre-cancerous [11-15]. If instituted at the right time, this would essentially either eliminate the need to use invasive techniques with adverse side effects (cone-biopsies, cauterizations, cryotherapies, chemo-/radio-therapy) or would at least supplement them [16,17]. In other words, it would offer a unique opportunity for medically treating women who did not receive the HPV-vaccine as girls and have never used potentially effective microbicidal agents such as carrageen [50,51], but have been diagnosed with pre-cancerous lesions at screening. However, this proposed mode of administration is not without its limitations, including uncertainties regarding the cells that will be targeted using the modeled 2xZFN-gene delivery and transduction, let alone the efficacy of ZFN-expression and or target-genome modification by the therapeutic proteins (ZFN_HpV_). Also, the delivery plasmids or vectors are potentially prone to attack by cells of the host immune-system, and concomitant local or systemic immunosuppressive (say steroidal) therapy might be needed to avoid this. Lastly, recent unbiased genome-wide analyses have shown that ZFNs may exhibit off-target effects *in-vivo* that might not be predicted by *in-silico* approaches such as the ones employed here [48,49].

In conclusion, we present databases of paired ZFAs targeting HPV-genomic-DNA (pZFA_HpV_) and their model ZFN_HpV_ for targeting two high-risk HPVs (types 16 and 18). With the appropriate optimization, modification of these modeled ZFN_HpV_ and* in-vivo *delivery HPV PsV encapsidation plasmids or vectors, a translational possibility of therapeutically reversing the primary HPV-induced oncogenic processes driving cervical neoplasia is proposed.

## Competing interests

WM is Chief Scientific Officer at Restrizymes Biotherapeutics (U) Ltd, and member of the steering committee of the Young and early careers’ investigators (YECI) of the Global HIV Vaccine Enterprise.

## Authors’ contribution

WM concieved the idea for this study, conducted the experiments, analysed the data, and wrote the final manuscript.

## Supplementary Material

Additional file 1 **List of zinc finger arrays targeting HPV type 16 genomic DNA.** This file offers a detailed list and loci of action of zinc finger arrays that specifically bind to 9 bp nucleotide sequences within the genomic DNA context of HPV type 16. Click here for file

Additional file 2 **List of zinc finger arrays binding to HPV type 18 genomic DNA.** This file offers a detailed list and loci of action of zinc finger arrays that specifically bind to 9 bp nucleotide sequences within the genomic DNA context of HPV type 18. Click here for file

Additional file 3 **List of zinc finger nucleases cleaving HPV type 16 genomic DNA.** This file offers a detailed list and loci of action of zinc finger nucleases that target and cleave >18 bp (9x2 + 5, 6, or 7) sequences within the genomic DNA context of HPV type 16. Click here for file

Additional file 4 **List of zinc finger nucleases cleaving HPV type 18 genomic DNA.** This file offers a detailed list and loci of action of zinc finger nucleases that target and cleave >18 bp (9x2 + 5, 6, or 7) sequences within the genomic DNA context of HPV type 18. Click here for file
