# Supplementary material for: Zinc finger arrays binding human papillomavirus types 16 and 18 genomic DNA: precursors of gene-therapeutics for in-situ reversal of associated cervical neoplasia
Source: Theor Biol Med Model. 2012 Jul 28;9:30. doi: 10.1186/1742-4682-9-30 (PMC3511202; doi:10.1186/1742-4682-9-30)
Supplement: Additional file 1 — List of zinc finger arrays targeting HPV type 16 genomic DNA. This file offers a detailed list and loci of action of zinc finger arrays that specifically bind to 9 bp nucleotide sequences within the genomic DNA context of HPV type 16. [file 1742-4682-9-30-S1.doc]

Single Zinc finger arrays (sZFAs) binding HPV type 16 genomic DNA

| **Zing finger array #**  (Target -DNA) | Recognition Helix | | (F1)(F2)(F3)-Array |
| --- | --- | --- | --- |
|  | **Finger:Helix** | **Triplets** |  |
| **ZFA-unknown-1**  40 t[tcggttacg](http://bindr.gdcb.iastate.edu:8080/ZiFDB/controller/searchArray?site=acggtttcg)c 30  40 aagccaatgcg 30 | F1: RSHDLTV | [ACG](http://bindr.gdcb.iastate.edu:8080/ZiFDB/controller/searchFinger?target=ACG) | (RSHDLTV)(HKSSLTR)(RADGLQL)- |
|  | F2: HKSSLTR | [GTT](http://bindr.gdcb.iastate.edu:8080/ZiFDB/controller/searchFinger?target=GTT) |  |
|  | F3: RADGLQL | [TCG](http://bindr.gdcb.iastate.edu:8080/ZiFDB/controller/searchFinger?target=TCG) |  |
| ZFA-unknown-2  41 a[tcggttgaa](http://bindr.gdcb.iastate.edu:8080/ZiFDB/controller/searchArray?site=gaagtttcg)c 51  41 tagccaacttg 51 | F1: QRSNLAR | [GAA](http://bindr.gdcb.iastate.edu:8080/ZiFDB/controller/searchFinger?target=GAA) | (QRSNLAR)(HKSSLTR)(RADGLQL)- |
|  | F2: HKSSLTR | [GTT](http://bindr.gdcb.iastate.edu:8080/ZiFDB/controller/searchFinger?target=GTT) |  |
|  | F3: RADGLQL | [TCG](http://bindr.gdcb.iastate.edu:8080/ZiFDB/controller/searchFinger?target=TCG) |  |
| **ZFA-unknown-3**  132 c[tgggtcgct](http://bindr.gdcb.iastate.edu:8080/ZiFDB/controller/searchArray?site=gctgtctgg)c 122  132 gacccagcgag 122 | F1: GATALKR | [GCT](http://bindr.gdcb.iastate.edu:8080/ZiFDB/controller/searchFinger?target=GCT) | (GATALKR)(DHSSLKR)(RSDHLSL)- |
|  | F2: DHSSLKR | [GTC](http://bindr.gdcb.iastate.edu:8080/ZiFDB/controller/searchFinger?target=GTC) |  |
|  | F3: RSDHLSL | [TGG](http://bindr.gdcb.iastate.edu:8080/ZiFDB/controller/searchFinger?target=TGG) |  |
| **ZFA-unknown-4**  145 c[tgtggtaac](http://bindr.gdcb.iastate.edu:8080/ZiFDB/controller/searchArray?site=aacggttgt)t 135  145 gacaccattga 135 | F1: GASALRS | [AAC](http://bindr.gdcb.iastate.edu:8080/ZiFDB/controller/searchFinger?target=AAC) | (GASALRS)(EAHHLSR)(QPHGLAH)- |
|  | F2: EAHHLSR | [GGT](http://bindr.gdcb.iastate.edu:8080/ZiFDB/controller/searchFinger?target=GGT) |  |
|  | F3: QPHGLAH | [TGT](http://bindr.gdcb.iastate.edu:8080/ZiFDB/controller/searchFinger?target=TGT) |  |
| **ZFA-unknown-5**  156 a[gctgcaaac](http://bindr.gdcb.iastate.edu:8080/ZiFDB/controller/searchArray?site=aacgcagct)a 166  156 tcgacgtttgt 166 | F1: GHTALRN | [AAC](http://bindr.gdcb.iastate.edu:8080/ZiFDB/controller/searchFinger?target=AAC) | (GHTALRN)(QSTTLKR)(LKHDLRR)- |
|  | F2: QSTTLKR | [GCA](http://bindr.gdcb.iastate.edu:8080/ZiFDB/controller/searchFinger?target=GCA) |  |
|  | F3: LKHDLRR | [GCT](http://bindr.gdcb.iastate.edu:8080/ZiFDB/controller/searchFinger?target=GCT) |  |
| **Details of ZFA-unknown-6 to ZFA-unknown-290 not shown** | | | |
| **ZFA-unknown-291**  7787 c[tagggtgac](http://bindr.gdcb.iastate.edu:8080/ZiFDB/controller/searchArray?site=gacggttag)a 7777  7787 gatcccactgt 7777 | F1: EEANLRR | [GAC](http://bindr.gdcb.iastate.edu:8080/ZiFDB/controller/searchFinger?target=GAC) | (EEANLRR)(EAHHLSR)(RRDNLPK)- |
|  | F2: EAHHLSR | [GGT](http://bindr.gdcb.iastate.edu:8080/ZiFDB/controller/searchFinger?target=GGT) |  |
|  | F3: RRDNLPK | [TAG](http://bindr.gdcb.iastate.edu:8080/ZiFDB/controller/searchFinger?target=TAG) |  |
| **ZFA-unknown-292**  7844 a[tgggtgtgt](http://bindr.gdcb.iastate.edu:8080/ZiFDB/controller/searchArray?site=tgtgtgtgg)g 7854  7844 tacccacacac 7854 | F1: RRQALEY | [TGT](http://bindr.gdcb.iastate.edu:8080/ZiFDB/controller/searchFinger?target=TGT) | (RRQALEY)(RREVLEN)(RRDHLSL)- |
|  | F2: RREVLEN | [GTG](http://bindr.gdcb.iastate.edu:8080/ZiFDB/controller/searchFinger?target=GTG) |  |
|  | F3: RRDHLSL | [TGG](http://bindr.gdcb.iastate.edu:8080/ZiFDB/controller/searchFinger?target=TGG) |  |
| **ZFA-unknown-293**  7850 g[tgtgcaaac](http://bindr.gdcb.iastate.edu:8080/ZiFDB/controller/searchArray?site=aacgcatgt)c 7860  7850 cacacgtttgg 7860 | F1: GHTALRN | [AAC](http://bindr.gdcb.iastate.edu:8080/ZiFDB/controller/searchFinger?target=AAC) | (GHTALRN)(QSTTLKR)(QAHGLTA)- |
|  | F2: QSTTLKR | [GCA](http://bindr.gdcb.iastate.edu:8080/ZiFDB/controller/searchFinger?target=GCA) |  |
|  | F3: QAHGLTA | [TGT](http://bindr.gdcb.iastate.edu:8080/ZiFDB/controller/searchFinger?target=TGT) |  |
| **ZFA-unknown-294**  7863 a[tcggtttgc](http://bindr.gdcb.iastate.edu:8080/ZiFDB/controller/searchArray?site=tgcgtttcg)a 7853  7863 tagccaaacgt 7853 | F1: RMRNLII | [TGC](http://bindr.gdcb.iastate.edu:8080/ZiFDB/controller/searchFinger?target=TGC) | (RMRNLII)(HKSSLTR)(RADGLQL)- |
|  | F2: HKSSLTR | [GTT](http://bindr.gdcb.iastate.edu:8080/ZiFDB/controller/searchFinger?target=GTT) |  |
|  | F3: RADGLQL | [TCG](http://bindr.gdcb.iastate.edu:8080/ZiFDB/controller/searchFinger?target=TCG) |  |
| **ZFA-unknown-295**  7889 a[gttgcttgt](http://bindr.gdcb.iastate.edu:8080/ZiFDB/controller/searchArray?site=tgtgctgtt)a 7879  7889 tcaacgaacat 7879 | F1: KRQHLEY | [TGT](http://bindr.gdcb.iastate.edu:8080/ZiFDB/controller/searchFinger?target=TGT) | (KRQHLEY)(QRSDLTR)(IRTSLKR)- |
|  | F2: QRSDLTR | [GCT](http://bindr.gdcb.iastate.edu:8080/ZiFDB/controller/searchFinger?target=GCT) |  |
|  | F3: IRTSLKR | [GTT](http://bindr.gdcb.iastate.edu:8080/ZiFDB/controller/searchFinger?target=GTT) |  |
| **ZFA-unknown-296**  7892 a[taagttgct](http://bindr.gdcb.iastate.edu:8080/ZiFDB/controller/searchArray?site=gctgtttaa)t 7882  7892 tattcaacgaa 7882 |  |  |  |
|  |  |  |  |
|  |  |  |  |
