# Supplementary material for: Zinc finger arrays binding human papillomavirus types 16 and 18 genomic DNA: precursors of gene-therapeutics for in-situ reversal of associated cervical neoplasia
Source: Theor Biol Med Model. 2012 Jul 28;9:30. doi: 10.1186/1742-4682-9-30 (PMC3511202; doi:10.1186/1742-4682-9-30)
Supplement: Additional file 2 — List of zinc finger arrays binding to HPV type 18 genomic DNA. This file offers a detailed list and loci of action of zinc finger arrays that specifically bind to 9 bp nucleotide sequences within the genomic DNA context of HPV type 18. [file 1742-4682-9-30-S2.doc]

Single zinc finger arrays (sZFAs) that recognize and bind HPV type 18 genomic DNA

| **Zing finger array #**  (Target -DNA) | Recognition patterns | | (F1)(F2)(F3)-Array |
| --- | --- | --- | --- |
|  | **Fingers:Helix** | **Triplets** |  |
| **ZFA-unknown-1**  99 t[gtggtgtgt](http://bindr.gdcb.iastate.edu:8080/ZiFDB/controller/searchArray?site=tgtgtggtg)t 89  99 acaccacacaa 89 | F1: RRQALEY | [TGT](http://bindr.gdcb.iastate.edu:8080/ZiFDB/controller/searchFinger?target=TGT) | (RRQALEY)(RREVLEN)(RKDALHV)- |
|  | F2: RREVLEN | [GTG](http://bindr.gdcb.iastate.edu:8080/ZiFDB/controller/searchFinger?target=GTG) |  |
|  | F3: RKDALHV | [GTG](http://bindr.gdcb.iastate.edu:8080/ZiFDB/controller/searchFinger?target=GTG) |  |
| **ZFA-unknown-2**  100 t[tgtggtgtg](http://bindr.gdcb.iastate.edu:8080/ZiFDB/controller/searchArray?site=gtgggttgt)t 90  100 aacaccacaca 90 | F1: RRFILSR | [GTG](http://bindr.gdcb.iastate.edu:8080/ZiFDB/controller/searchFinger?target=GTG) | (RRFILSR)(EAHHLSR)(QPHGLAH)- |
|  | F2: EAHHLSR | [GGT](http://bindr.gdcb.iastate.edu:8080/ZiFDB/controller/searchFinger?target=GGT) |  |
|  | F3: QPHGLAH | [TGT](http://bindr.gdcb.iastate.edu:8080/ZiFDB/controller/searchFinger?target=TGT) |  |
| **ZFA-unknown-3**  136 g[gtcgccgtg](http://bindr.gdcb.iastate.edu:8080/ZiFDB/controller/searchArray?site=gtggccgtc)t 126  136 ccagcggcaca 126 | F1: RTSSLKR | [GTG](http://bindr.gdcb.iastate.edu:8080/ZiFDB/controller/searchFinger?target=GTG) | (RTSSLKR)(DSSVLRR)(EGGALRR)- |
|  | F2: DSSVLRR | [GCC](http://bindr.gdcb.iastate.edu:8080/ZiFDB/controller/searchFinger?target=GCC) |  |
|  | F3: EGGALRR | [GTC](http://bindr.gdcb.iastate.edu:8080/ZiFDB/controller/searchFinger?target=GTC) |  |
| **ZFA-unknown-4**  141 t[gtagggtcg](http://bindr.gdcb.iastate.edu:8080/ZiFDB/controller/searchArray?site=tcgggggta)c 131  141 acatcccagcg 131 | F1: KNNDLLK | [TCG](http://bindr.gdcb.iastate.edu:8080/ZiFDB/controller/searchFinger?target=TCG) | (KNNDLLK)(EAHHLSR)(QSTSLQR)- |
|  | F2: EAHHLSR | [GGG](http://bindr.gdcb.iastate.edu:8080/ZiFDB/controller/searchFinger?target=GGG) |  |
|  | F3: QSTSLQR | [GTA](http://bindr.gdcb.iastate.edu:8080/ZiFDB/controller/searchFinger?target=GTA) |  |
| **ZFA-unknown-5**  148 g[gtagcttgt](http://bindr.gdcb.iastate.edu:8080/ZiFDB/controller/searchArray?site=tgtgctgta)a 138  148 ccatcgaacat 138 | F1: KRQHLEY | [TGT](http://bindr.gdcb.iastate.edu:8080/ZiFDB/controller/searchFinger?target=TGT) | (KRQHLEY)(QRSDLTR)(QSGTLTR)- |
|  | F2: QRSDLTR | [GCT](http://bindr.gdcb.iastate.edu:8080/ZiFDB/controller/searchFinger?target=GCT) |  |
|  | F3: QSGTLTR | [GTA](http://bindr.gdcb.iastate.edu:8080/ZiFDB/controller/searchFinger?target=GCT) |  |
| **Details of ZFA-unknown-6 to ZFA-unknown-321 not shown** | | | |
| **ZFA-unknown-322**  7748 a[gtagttgta](http://bindr.gdcb.iastate.edu:8080/ZiFDB/controller/searchArray?site=gtagttgta)c 7738  7748 tcatcaacatg 7738 | F1: QQQALKR | [GTA](http://bindr.gdcb.iastate.edu:8080/ZiFDB/controller/searchFinger?target=GTA) | (QQQALKR)(HKSSLTR)(QSTSLQR)- |
|  | F2: HKSSLTR | [GTT](http://bindr.gdcb.iastate.edu:8080/ZiFDB/controller/searchFinger?target=GTT) |  |
|  | F3: QSTSLQR | [GTA](http://bindr.gdcb.iastate.edu:8080/ZiFDB/controller/searchFinger?target=GTA) |  |
| **ZFA-unknown-323**  7795 c[taagctgtg](http://bindr.gdcb.iastate.edu:8080/ZiFDB/controller/searchArray?site=gtggcttaa)c 7805  7795 gattcgacacg 7805 | F1: RTSSLKR | [GTG](http://bindr.gdcb.iastate.edu:8080/ZiFDB/controller/searchFinger?target=GTG) | (RTSSLKR)(QRSDLTR)(QRGNLNM)- |
|  | F2: QRSDLTR | [GCT](http://bindr.gdcb.iastate.edu:8080/ZiFDB/controller/searchFinger?target=GCT) |  |
|  | F3: QRGNLNM | [TAA](http://bindr.gdcb.iastate.edu:8080/ZiFDB/controller/searchFinger?target=TAA) |  |
| **ZFA-unknown-324**  7827 t[tcggttgca](http://bindr.gdcb.iastate.edu:8080/ZiFDB/controller/searchArray?site=gcagtttcg)t 7817  7827 aagccaacgta 7817 | F1: RRQELKR | [GCA](http://bindr.gdcb.iastate.edu:8080/ZiFDB/controller/searchFinger?target=GCA) | (RRQELKR)(HKSSLTR)(RADGLQL)- |
|  | F2: HKSSLTR | [GTT](http://bindr.gdcb.iastate.edu:8080/ZiFDB/controller/searchFinger?target=GTT) |  |
|  | F3: RADGLQL | [TCG](http://bindr.gdcb.iastate.edu:8080/ZiFDB/controller/searchFinger?target=TCG) |  |
| **ZFA-unknown-325**  7828 a[taggttggg](http://bindr.gdcb.iastate.edu:8080/ZiFDB/controller/searchArray?site=ggggtttag)c 7838  7828 tatccaacccg 7838 | F1: KSNHLHV | [GGG](http://bindr.gdcb.iastate.edu:8080/ZiFDB/controller/searchFinger?target=GGG) | (KSNHLHV)(HKSSLTR)(RPESLAP)- |
|  | F2: HKSSLTR | [GTT](http://bindr.gdcb.iastate.edu:8080/ZiFDB/controller/searchFinger?target=GTT) |  |
|  | F3: RPESLAP | [TAG](http://bindr.gdcb.iastate.edu:8080/ZiFDB/controller/searchFinger?target=TAG) |  |
| **ZFA-unknown-326**  7833 t[tgggcagca](http://bindr.gdcb.iastate.edu:8080/ZiFDB/controller/searchArray?site=gcagcatgg)c 7843  7833 aacccgtcgtg 7843 | F1: QRGTLNR | [GCA](http://bindr.gdcb.iastate.edu:8080/ZiFDB/controller/searchFinger?target=GCA) | (QRGTLNR)(QSTTLKR)(RSDHLSL)- |
|  | F2: QSTTLKR | [GCA](http://bindr.gdcb.iastate.edu:8080/ZiFDB/controller/searchFinger?target=GCA) |  |
|  | F3: RSDHLSL | [TGG](http://bindr.gdcb.iastate.edu:8080/ZiFDB/controller/searchFinger?target=TGG) |  |
| **ZFA-unknown-327**  7845 a[tgtgctgcc](http://bindr.gdcb.iastate.edu:8080/ZiFDB/controller/searchArray?site=gccgcttgt)c 7835  7845 tacacgacggg 7835 |  |  |  |
|  |  |  |  |
|  |  |  |  |
