# Supplementary material for: Zinc finger arrays binding human papillomavirus types 16 and 18 genomic DNA: precursors of gene-therapeutics for in-situ reversal of associated cervical neoplasia
Source: Theor Biol Med Model. 2012 Jul 28;9:30. doi: 10.1186/1742-4682-9-30 (PMC3511202; doi:10.1186/1742-4682-9-30)
Supplement: Additional file 3 — List of zinc finger nucleases cleaving HPV type 16 genomic DNA. This file offers a detailed list and loci of action of zinc finger nucleases that target and cleave >18 bp (9x2 + 5, 6, or 7) sequences within the genomic DNA context of HPV type 16. [file 1742-4682-9-30-S3.doc]

Paired Zinc finger arrays (pZFA) for engineering nucleases targeting and cleaving sequences within the HPV-16 genome

| **Zing finger nuclease-spacer- #**  (Target –genomic context) | **Recognition Helix**  (Target-DNA) | |
| --- | --- | --- |
|  | **L-Finger** | **R-Finger** |
| **ZFN-unknown-SP-7-1** 3413 ccgcgacccataccaaa[gccgtcgcc](http://bindr.gdcb.iastate.edu:8080/ZiFDB/controller/searchArray?site=gccgtcgcc)t 3439  3413 g[gcgctgggt](http://bindr.gdcb.iastate.edu:8080/ZiFDB/controller/searchArray?site=gcggtctgg)atggtttcggcagcgga 3439 | F1: RRHGLDR | F1: DSPTLRR |
|  | F2: DHSSLKR | F2: DHSSLKR |
|  | F3: RSDHLSL | F3: DPSNLRR |
|  | [GCG](http://bindr.gdcb.iastate.edu:8080/ZiFDB/controller/searchFinger?target=GCG)[GTC](http://bindr.gdcb.iastate.edu:8080/ZiFDB/controller/searchFinger?target=GTC)[TGG](http://bindr.gdcb.iastate.edu:8080/ZiFDB/controller/searchFinger?target=TGG) | [GCC](http://bindr.gdcb.iastate.edu:8080/ZiFDB/controller/searchFinger?target=GCC)[GTC](http://bindr.gdcb.iastate.edu:8080/ZiFDB/controller/searchFinger?target=GTC)[GCC](http://bindr.gdcb.iastate.edu:8080/ZiFDB/controller/searchFinger?target=GCC) |
| **ZFN-unknown-SP-5-1** 3501 cccctgccacaccac[taagttgtt](http://bindr.gdcb.iastate.edu:8080/ZiFDB/controller/searchArray?site=gttgtttaa)g 3525  3501 g[gggacggtg](http://bindr.gdcb.iastate.edu:8080/ZiFDB/controller/searchArray?site=ggggcagtg)tggtgattcaacaac 3525 | F1: RRAHLQN | F1: TKPVLKI |
|  | F2: QSTTLKR | F2: HKSSLTR |
|  | F3: RKDALHV | F3: QQGNLQL |
|  | [GGG](http://bindr.gdcb.iastate.edu:8080/ZiFDB/controller/searchFinger?target=GGG)[GCA](http://bindr.gdcb.iastate.edu:8080/ZiFDB/controller/searchFinger?target=GCA)[GTG](http://bindr.gdcb.iastate.edu:8080/ZiFDB/controller/searchFinger?target=GTG) | [TAA](http://bindr.gdcb.iastate.edu:8080/ZiFDB/controller/searchFinger?target=TAA)[GTTGTT](http://bindr.gdcb.iastate.edu:8080/ZiFDB/controller/searchFinger?target=GTT) |
| **ZFN-unknown-SP-5-2** 5920 acacagcggctggtt[tgggcctgt](http://bindr.gdcb.iastate.edu:8080/ZiFDB/controller/searchArray?site=tgtgcctgg)g 5944  5920 t[gtgtcgccg](http://bindr.gdcb.iastate.edu:8080/ZiFDB/controller/searchArray?site=gtggctgcc)accaaacccggacac 5944 | F1: RTSSLKR | F1: RKQHLTL |
|  | F2: QRSDLTR | F2: DSSVLRR |
|  | F3: DPSNLRR | F3: RSDHLSL |
|  | [GTG](http://bindr.gdcb.iastate.edu:8080/ZiFDB/controller/searchFinger?target=GTG)[GCT](http://bindr.gdcb.iastate.edu:8080/ZiFDB/controller/searchFinger?target=GCT)[GCC](http://bindr.gdcb.iastate.edu:8080/ZiFDB/controller/searchFinger?target=GCC) | [TGG](http://bindr.gdcb.iastate.edu:8080/ZiFDB/controller/searchFinger?target=TGG)[GCC](http://bindr.gdcb.iastate.edu:8080/ZiFDB/controller/searchFinger?target=GCC)[TGT](http://bindr.gdcb.iastate.edu:8080/ZiFDB/controller/searchFinger?target=TGT) |
| **ZFN-unknown-SP-7-2** 6929 cacctccagcacctaaa[gaagatgat](http://bindr.gdcb.iastate.edu:8080/ZiFDB/controller/searchArray?site=gatgatgaa)c 6955  6929 g[tggaggtcg](http://bindr.gdcb.iastate.edu:8080/ZiFDB/controller/searchArray?site=ggtggagct)tggatttcttctactag 6955 | F1: IPNHLAR | F1: TKQRLVV |
|  | F2: QSAHLKR | F2: VRHNLTR |
|  | F3: LKHDLRR | F3: QRNNLGR |
|  | [GGT](http://bindr.gdcb.iastate.edu:8080/ZiFDB/controller/searchFinger?target=GGT)[GGA](http://bindr.gdcb.iastate.edu:8080/ZiFDB/controller/searchFinger?target=GGA)[GCT](http://bindr.gdcb.iastate.edu:8080/ZiFDB/controller/searchFinger?target=GCT) | [GAA](http://bindr.gdcb.iastate.edu:8080/ZiFDB/controller/searchFinger?target=GAA)[GATGAT](http://bindr.gdcb.iastate.edu:8080/ZiFDB/controller/searchFinger?target=GAT) |
| **ZFN-unknown-SP-7-3** 7129 gctaaacgcaaaaaacg[taagctgta](http://bindr.gdcb.iastate.edu:8080/ZiFDB/controller/searchArray?site=gtagcttaa)a 7155  7129 c[gatttgcgt](http://bindr.gdcb.iastate.edu:8080/ZiFDB/controller/searchArray?site=taggtttgc)tttttgcattcgacatt 7155 | F1: RSHNLRL | F1: QQSSLLR |
|  | F2: HKSSLTR | F2: QRSDLTR |
|  | F3: QNRSLAH | F3: QRGNLNM |
|  | [TAG](http://bindr.gdcb.iastate.edu:8080/ZiFDB/controller/searchFinger?target=TAG)[GTT](http://bindr.gdcb.iastate.edu:8080/ZiFDB/controller/searchFinger?target=GTT)[TGC](http://bindr.gdcb.iastate.edu:8080/ZiFDB/controller/searchFinger?target=TGC) | [TAA](http://bindr.gdcb.iastate.edu:8080/ZiFDB/controller/searchFinger?target=TAA)[GCT](http://bindr.gdcb.iastate.edu:8080/ZiFDB/controller/searchFinger?target=GCT)[GTA](http://bindr.gdcb.iastate.edu:8080/ZiFDB/controller/searchFinger?target=GTA) |
| **ZFN-unknown-SP-7-4** 7333 tttcaacacctactaat[tgtgttgtg](http://bindr.gdcb.iastate.edu:8080/ZiFDB/controller/searchArray?site=gtggtttgt)g 7359  7333 a[aagttgtgg](http://bindr.gdcb.iastate.edu:8080/ZiFDB/controller/searchArray?site=gaagttggt)atgattaacacaacacc 7359 | F1: QRSNLAR | F1: RNFILQR |
|  | F2: HKSSLTR | F2: HKSSLTR |
|  | F3: HGHRLKT | F3: QPHGLAH |
|  | [GAA](http://bindr.gdcb.iastate.edu:8080/ZiFDB/controller/searchFinger?target=GAA)[GTT](http://bindr.gdcb.iastate.edu:8080/ZiFDB/controller/searchFinger?target=GTT)[GGT](http://bindr.gdcb.iastate.edu:8080/ZiFDB/controller/searchFinger?target=GGT) | [TGT](http://bindr.gdcb.iastate.edu:8080/ZiFDB/controller/searchFinger?target=TGT)[GTT](http://bindr.gdcb.iastate.edu:8080/ZiFDB/controller/searchFinger?target=GTT)[GTG](http://bindr.gdcb.iastate.edu:8080/ZiFDB/controller/searchFinger?target=GTG) |
| **ZFN-unknown-SP-7-5** 7336 caacacctactaattgt[gttgtggtt](http://bindr.gdcb.iastate.edu:8080/ZiFDB/controller/searchArray?site=gttgtggtt)a 7362  7336 g[ttgtggatg](http://bindr.gdcb.iastate.edu:8080/ZiFDB/controller/searchArray?site=gttggtgta)attaacacaacaccaat 7362 | F1: AATALRR | F1: MNSVLKR |
|  | F2: EAHHLSR | F2: RREVLEN |
|  | F3: QSTSLQR | F3: INHSLRR |
|  | [GTT](http://bindr.gdcb.iastate.edu:8080/ZiFDB/controller/searchFinger?target=GTT)[GGT](http://bindr.gdcb.iastate.edu:8080/ZiFDB/controller/searchFinger?target=GGT)[GTA](http://bindr.gdcb.iastate.edu:8080/ZiFDB/controller/searchFinger?target=GTA) | [GTT](http://bindr.gdcb.iastate.edu:8080/ZiFDB/controller/searchFinger?target=GTT)[GTG](http://bindr.gdcb.iastate.edu:8080/ZiFDB/controller/searchFinger?target=GTG)[GTT](http://bindr.gdcb.iastate.edu:8080/ZiFDB/controller/searchFinger?target=GTT) |
| **ZFN-unknown-SP-5-3** 7443 tagcttcaaccgaat[tcggttgca](http://bindr.gdcb.iastate.edu:8080/ZiFDB/controller/searchArray?site=gcagtttcg)t 7467  7443 a[tcgaagttg](http://bindr.gdcb.iastate.edu:8080/ZiFDB/controller/searchArray?site=gctgaagtt)gcttaagccaacgta 7467 | F1: QRQALDR | F1: RRQELKR |
|  | F2: QQTNLTR | F2: HKSSLTR |
|  | F3: VGGSLNR | F3: RADGLQL |
|  | [GCT](http://bindr.gdcb.iastate.edu:8080/ZiFDB/controller/searchFinger?target=GCT)[GAA](http://bindr.gdcb.iastate.edu:8080/ZiFDB/controller/searchFinger?target=GAA)[GTT](http://bindr.gdcb.iastate.edu:8080/ZiFDB/controller/searchFinger?target=GTT) | [TCG](http://bindr.gdcb.iastate.edu:8080/ZiFDB/controller/searchFinger?target=TCG)[GTT](http://bindr.gdcb.iastate.edu:8080/ZiFDB/controller/searchFinger?target=GTT)[GCA](http://bindr.gdcb.iastate.edu:8080/ZiFDB/controller/searchFinger?target=GCA) |
| **ZFN-unknown-SP-5-4** 7446 cttcaaccgaattcg[gttgcatgc](http://bindr.gdcb.iastate.edu:8080/ZiFDB/controller/searchArray?site=tgcgcagtt)t 7470  7446 g[aagttggct](http://bindr.gdcb.iastate.edu:8080/ZiFDB/controller/searchArray?site=gaagtttcg)taagccaacgtacga 7470 |  |  |
|  |  |  |
|  |  |  |
|  |  |  |
