# Supplementary material for: Zinc finger arrays binding human papillomavirus types 16 and 18 genomic DNA: precursors of gene-therapeutics for in-situ reversal of associated cervical neoplasia
Source: Theor Biol Med Model. 2012 Jul 28;9:30. doi: 10.1186/1742-4682-9-30 (PMC3511202; doi:10.1186/1742-4682-9-30)
Supplement: Additional file 4 — List of zinc finger nucleases cleaving HPV type 18 genomic DNA. This file offers a detailed list and loci of action of zinc finger nucleases that target and cleave >18 bp (9x2 + 5, 6, or 7) sequences within the genomic DNA context of HPV type 18. [file 1742-4682-9-30-S4.doc]

Paired Zinc finger arrays (pZFA) for engineering nucleases targeting and cleaving sequences within the HPV-18 genome

| **Zing finger nuclease-sapcer- #**  (Target –genomic context) | **Recognition Helix**  (Target-DNA) | |
| --- | --- | --- |
|  | **L-Finger** | **R-Finger** |
| **ZFN-unknown-SP-6-1** 758 caacgtcacacaatgt[tgtgtatgt](http://bindr.gdcb.iastate.edu:8080/ZiFDB/controller/searchArray?site=tgtgtatgt)g 783  758 g[ttgcagtgt](http://bindr.gdcb.iastate.edu:8080/ZiFDB/controller/searchArray?site=gttgactgt)gttacaacacatacac 783 | F1: THTVLAR | F1: RKQHLQL |
|  | F2: DRGNLTR | F2: QRSSLVR |
|  | F3: QPHGLRA | F3: QPHGLAH |
|  | [GTT](http://bindr.gdcb.iastate.edu:8080/ZiFDB/controller/searchFinger?target=GTT)[GAC](http://bindr.gdcb.iastate.edu:8080/ZiFDB/controller/searchFinger?target=GAC)[TGT](http://bindr.gdcb.iastate.edu:8080/ZiFDB/controller/searchFinger?target=TGT) | [TGT](http://bindr.gdcb.iastate.edu:8080/ZiFDB/controller/searchFinger?target=TGT)[GTA](http://bindr.gdcb.iastate.edu:8080/ZiFDB/controller/searchFinger?target=GTA)[TGT](http://bindr.gdcb.iastate.edu:8080/ZiFDB/controller/searchFinger?target=TGT) |
| **ZFN-unknown-SP-6-2** 1853 attcaaccaccaaaat[tgcgaagta](http://bindr.gdcb.iastate.edu:8080/ZiFDB/controller/searchArray?site=gtagaatgc)g 1878  1853 t[aagttggtg](http://bindr.gdcb.iastate.edu:8080/ZiFDB/controller/searchArray?site=gaagttgtg)gttttaacgcttcatc 1878 | F1: QRSNLAR | F1: QKQALDR |
|  | F2: HKSSLTR | F2: QQTNLTR |
|  | F3: RNTALQH | F4: ANRTLVH |
|  | [GAA](http://bindr.gdcb.iastate.edu:8080/ZiFDB/controller/searchFinger?target=GAA)[GTT](http://bindr.gdcb.iastate.edu:8080/ZiFDB/controller/searchFinger?target=GTT)[GTG](http://bindr.gdcb.iastate.edu:8080/ZiFDB/controller/searchFinger?target=GTG) | [TGC](http://bindr.gdcb.iastate.edu:8080/ZiFDB/controller/searchFinger?target=TGC)[GAA](http://bindr.gdcb.iastate.edu:8080/ZiFDB/controller/searchFinger?target=GAA)[GTA](http://bindr.gdcb.iastate.edu:8080/ZiFDB/controller/searchFinger?target=GTA) |
| **ZFN-unknown-SP-6-3** 2083 agacagcaacagcaat[gcagctgcc](http://bindr.gdcb.iastate.edu:8080/ZiFDB/controller/searchArray?site=gccgctgca)t 2108  2083 t[ctgtcgttg](http://bindr.gdcb.iastate.edu:8080/ZiFDB/controller/searchArray?site=gtcgctgtt)tcgttacgtcgacgga 2108 | F1: TRAVLRR | F1: DRRTLDR |
|  | F2: QRSDLTR | F2: QRSDLTR |
|  | F3: IRTSLKR | F3: QGGTLRR |
|  | [GTC](http://bindr.gdcb.iastate.edu:8080/ZiFDB/controller/searchFinger?target=GTC)[GCT](http://bindr.gdcb.iastate.edu:8080/ZiFDB/controller/searchFinger?target=GCT)[GTT](http://bindr.gdcb.iastate.edu:8080/ZiFDB/controller/searchFinger?target=GTT) | [GCA](http://bindr.gdcb.iastate.edu:8080/ZiFDB/controller/searchFinger?target=GCA)[GCT](http://bindr.gdcb.iastate.edu:8080/ZiFDB/controller/searchFinger?target=GCT)[GCC](http://bindr.gdcb.iastate.edu:8080/ZiFDB/controller/searchFinger?target=GCC) |
| **ZFN-unknown-SP-6-4** 3530 gacctacggccagacg[tcggctgct](http://bindr.gdcb.iastate.edu:8080/ZiFDB/controller/searchArray?site=gctgcttcg)a 3555  3530 c[tggatgccg](http://bindr.gdcb.iastate.edu:8080/ZiFDB/controller/searchArray?site=ggtgtagcc)gtctgcagccgacgat 3555 | F1: TRTRLVI | F1: MKNTLTR |
|  | F2: QRSSLVR | F2: QRSDLTR |
|  | F3: DKSVLAR | F3: RSDGLRG |
|  | [GGT](http://bindr.gdcb.iastate.edu:8080/ZiFDB/controller/searchFinger?target=GGT)[GTA](http://bindr.gdcb.iastate.edu:8080/ZiFDB/controller/searchFinger?target=GTA)[GCC](http://bindr.gdcb.iastate.edu:8080/ZiFDB/controller/searchFinger?target=GCC) | [TCG](http://bindr.gdcb.iastate.edu:8080/ZiFDB/controller/searchFinger?target=TCG)[GCTGCT](http://bindr.gdcb.iastate.edu:8080/ZiFDB/controller/searchFinger?target=GCT) |
| **ZFN-unknown-SP-6-5** 3602 tgtcaacccacttctc[ggtgcagct](http://bindr.gdcb.iastate.edu:8080/ZiFDB/controller/searchArray?site=gctgcaggt)a 3627  3602 a[cagttgggt](http://bindr.gdcb.iastate.edu:8080/ZiFDB/controller/searchArray?site=gacgtttgg)gaagagccacgtcgat 3627 | F1: EQANLRR | F1: TKQILGR |
|  | F2: HKSSLTR | F2: QSTTLKR |
|  | F3: RSDHLSL | F3: VDHHLRR |
|  | [GAC](http://bindr.gdcb.iastate.edu:8080/ZiFDB/controller/searchFinger?target=GAC)[GTT](http://bindr.gdcb.iastate.edu:8080/ZiFDB/controller/searchFinger?target=GTT)[TGG](http://bindr.gdcb.iastate.edu:8080/ZiFDB/controller/searchFinger?target=TGG) | [G](http://bindr.gdcb.iastate.edu:8080/ZiFDB/controller/searchFinger?target=GGT)[GCA](http://bindr.gdcb.iastate.edu:8080/ZiFDB/controller/searchFinger?target=GCA)GT[GCT](http://bindr.gdcb.iastate.edu:8080/ZiFDB/controller/searchFinger?target=GCT) |
| **ZFN-unknown-SP-7-1** 3632 tacaggcaacaacaaaa[gacggaaac](http://bindr.gdcb.iastate.edu:8080/ZiFDB/controller/searchArray?site=aacggagac)t 3658  3632 a[tgtccgttg](http://bindr.gdcb.iastate.edu:8080/ZiFDB/controller/searchArray?site=tgtgccgtt)ttgttttctgcctttga 3658 | F1: RKQHLTL | F1: GGTALVM |
|  | F2: DSSVLRR | F2: QSAHLKR |
|  | F3: IRTSLKR | F3: DPSNLRR |
|  | [TGT](http://bindr.gdcb.iastate.edu:8080/ZiFDB/controller/searchFinger?target=TGT)[GCC](http://bindr.gdcb.iastate.edu:8080/ZiFDB/controller/searchFinger?target=GCC)[GTT](http://bindr.gdcb.iastate.edu:8080/ZiFDB/controller/searchFinger?target=GTT) | [GAC](http://bindr.gdcb.iastate.edu:8080/ZiFDB/controller/searchFinger?target=GAC)[GGA](http://bindr.gdcb.iastate.edu:8080/ZiFDB/controller/searchFinger?target=GGA)[AAC](http://bindr.gdcb.iastate.edu:8080/ZiFDB/controller/searchFinger?target=AAC) |
| **ZFN-unknown-SP-6-6** 4842 ttgcttcttctggtac[gggggagga](http://bindr.gdcb.iastate.edu:8080/ZiFDB/controller/searchArray?site=ggaggaggg)a 4867  4842 a[acgaagaag](http://bindr.gdcb.iastate.edu:8080/ZiFDB/controller/searchArray?site=gcagaagaa)accatgccccctcctt 4867 | F1: RGQELRR | F1: RTDRLIR |
|  | F2: QQTNLTR | F2: QSAHLKR |
|  | F3: QTNNLNR | F3: RTEHLAR |
|  | [GCA](http://bindr.gdcb.iastate.edu:8080/ZiFDB/controller/searchFinger?target=GCA)[GAAGAA](http://bindr.gdcb.iastate.edu:8080/ZiFDB/controller/searchFinger?target=GAA) | [GGG](http://bindr.gdcb.iastate.edu:8080/ZiFDB/controller/searchFinger?target=GGG)[GGAGGA](http://bindr.gdcb.iastate.edu:8080/ZiFDB/controller/searchFinger?target=GGA) |
| **ZFN-unknown-SP-7-2** 4842 ttgcttcttctggtacg[ggggaggaa](http://bindr.gdcb.iastate.edu:8080/ZiFDB/controller/searchArray?site=gaagagggg)c 4868  4842 a[acgaagaag](http://bindr.gdcb.iastate.edu:8080/ZiFDB/controller/searchArray?site=gcagaagaa)accatgccccctccttg 4868 | F1: RGQELRR | F1: QASNLLR |
|  | F2: QQTNLTR | F2: RQDNLGR |
|  | F3: QTNNLNR | F3: RIDKLGG |
|  | [GCA](http://bindr.gdcb.iastate.edu:8080/ZiFDB/controller/searchFinger?target=GCA)[GAAGAA](http://bindr.gdcb.iastate.edu:8080/ZiFDB/controller/searchFinger?target=GAA) | [GGG](http://bindr.gdcb.iastate.edu:8080/ZiFDB/controller/searchFinger?target=GGG)[GAG](http://bindr.gdcb.iastate.edu:8080/ZiFDB/controller/searchFinger?target=GAG)[GAA](http://bindr.gdcb.iastate.edu:8080/ZiFDB/controller/searchFinger?target=GAA) |
| **ZFN-unknown-SP-6-7** 5415 taacctcctcttggga[tgtgcctgt](http://bindr.gdcb.iastate.edu:8080/ZiFDB/controller/searchArray?site=tgtgcctgt)a 5440  5415 a[ttggaggag](http://bindr.gdcb.iastate.edu:8080/ZiFDB/controller/searchArray?site=gttgaggag)aaccctacacggacat 5440 | F1: TTTVLAR | F1: RKQHLTL |
|  | F2: RQDNLGR | F2: DSSVLRR |
|  | F3: RVDNLPR | F3: QAHGLTA |
|  | [GTT](http://bindr.gdcb.iastate.edu:8080/ZiFDB/controller/searchFinger?target=GTT)[GAGGAG](http://bindr.gdcb.iastate.edu:8080/ZiFDB/controller/searchFinger?target=GAG) | [TGT](http://bindr.gdcb.iastate.edu:8080/ZiFDB/controller/searchFinger?target=TGT)[GCC](http://bindr.gdcb.iastate.edu:8080/ZiFDB/controller/searchFinger?target=GCC)[TGT](http://bindr.gdcb.iastate.edu:8080/ZiFDB/controller/searchFinger?target=TGT) |
| **ZFN-unknown-SP-7-3** 6013 ccgccacgtctaatgtt[tctgaggac](http://bindr.gdcb.iastate.edu:8080/ZiFDB/controller/searchArray?site=gacgagtct)g 6039  6013 g[gcggtgcag](http://bindr.gdcb.iastate.edu:8080/ZiFDB/controller/searchArray?site=gcggtggac)attacaaagactcctgc 6039 | F1: RAHTLRR | F1: DEANLRR |
|  | F2: RREVLEN | F2: RQDNLGR |
|  | F3: DPSNLRR | F3: QRNTLKG |
|  | [GCG](http://bindr.gdcb.iastate.edu:8080/ZiFDB/controller/searchFinger?target=GCG)[GTG](http://bindr.gdcb.iastate.edu:8080/ZiFDB/controller/searchFinger?target=GTG)[GAC](http://bindr.gdcb.iastate.edu:8080/ZiFDB/controller/searchFinger?target=GAC) | [TCT](http://bindr.gdcb.iastate.edu:8080/ZiFDB/controller/searchFinger?target=TCT)[GAG](http://bindr.gdcb.iastate.edu:8080/ZiFDB/controller/searchFinger?target=GAG)[GAC](http://bindr.gdcb.iastate.edu:8080/ZiFDB/controller/searchFinger?target=GAC) |
| **ZFN-unknown-SP-5-1** 6457 tgcctgcttcacctg[gcagctgtg](http://bindr.gdcb.iastate.edu:8080/ZiFDB/controller/searchArray?site=gtggctgca)t 6481  6457 a[cggacgaag](http://bindr.gdcb.iastate.edu:8080/ZiFDB/controller/searchArray?site=ggcgcagaa)tggaccgtcgacaca 6481 | F1: VPSKLLR | F1: RTSSLKR |
|  | F2: QSTTLKR | F2: QRSDLTR |
|  | F3: QRNNLGR | F3: QGGTLRR |
|  | [GGC](http://bindr.gdcb.iastate.edu:8080/ZiFDB/controller/searchFinger?target=GGC)[GCA](http://bindr.gdcb.iastate.edu:8080/ZiFDB/controller/searchFinger?target=GCA)[GAA](http://bindr.gdcb.iastate.edu:8080/ZiFDB/controller/searchFinger?target=GAA) | [GCA](http://bindr.gdcb.iastate.edu:8080/ZiFDB/controller/searchFinger?target=GCA)[GCT](http://bindr.gdcb.iastate.edu:8080/ZiFDB/controller/searchFinger?target=GCT)[GTG](http://bindr.gdcb.iastate.edu:8080/ZiFDB/controller/searchFinger?target=GTG) |
| **ZFN-unknown-SP-5-2** 6460 ctgcttcacctggca[gctgtgtgt](http://bindr.gdcb.iastate.edu:8080/ZiFDB/controller/searchArray?site=tgtgtggct)a 6484  6460 g[acgaagtgg](http://bindr.gdcb.iastate.edu:8080/ZiFDB/controller/searchArray?site=gcagaaggt)accgtcgacacacat 6484 | F1: RGQELRR | F1: RRQALEY |
|  | F2: QQTNLTR | F2: RREVLEN |
|  | F3: IRHHLKR | F3: VGASLKR |
|  | [GCA](http://bindr.gdcb.iastate.edu:8080/ZiFDB/controller/searchFinger?target=GCA)[GAA](http://bindr.gdcb.iastate.edu:8080/ZiFDB/controller/searchFinger?target=GAA)[GGT](http://bindr.gdcb.iastate.edu:8080/ZiFDB/controller/searchFinger?target=GGT) | [GCT](http://bindr.gdcb.iastate.edu:8080/ZiFDB/controller/searchFinger?target=GCT)[GTG](http://bindr.gdcb.iastate.edu:8080/ZiFDB/controller/searchFinger?target=GTG)[TGT](http://bindr.gdcb.iastate.edu:8080/ZiFDB/controller/searchFinger?target=TGT) |
| **ZFN-unknown-SP-7-4** 7817 atgcaaccgaaataggt[tgggcagca](http://bindr.gdcb.iastate.edu:8080/ZiFDB/controller/searchArray?site=gcagcatgg)c 7843  7817 t[acgttggct](http://bindr.gdcb.iastate.edu:8080/ZiFDB/controller/searchArray?site=gcagtttcg)ttatccaacccgtcgtg 7843 |  |  |
|  |  |  |
|  |  |  |
|  |  |  |
